# Net absorption and liver metabolism of amino acids and heat production of portal-drained viscera and liver in multiparous sows during transition and lactation

**DOI:** 10.1186/s40104-019-0417-7

**Published:** 2020-02-12

**Authors:** Liang Hu, Niels Bastian Kristensen, Lianqiang Che, De Wu, Peter Kappel Theil

**Affiliations:** 1 Key Laboratory for Animal Disease-Resistance Nutrition of China Ministry of Education, Institute of Animal Nutrition, Sichuan Agricultural University, No. 211, Huimin Road, Wenjiang District, Chengdu, Sichuan 611130 People’s Republic of China; 2 Department of Animal Science, Faculty of Science and Technology, Aarhus University, DK-8830 Tjele, Denmark; 3 Present address: SEGES Danish Pig Research Centre, DK-1609 Copenhagen, Denmark

**Keywords:** Gestation, Heat production, Lactation, Net hepatic flux, Net portal flux, Sow

## Abstract

**Background:**

Determination of nutrient requirements in the late gestating and lactating sows is essential to optimize sow productivity. The objectives of the present study were to quantify amino acid (AA) fluxes and heat production across portal-drained viscera (PDV) and liver in multiparous sows during transition and lactation.

**Methods:**

Eight second parity sows were fitted with indwelling catheters in the femoral artery and in the mesenteric, portal and hepatic veins. Eight hourly sets of blood samples were taken starting 0.5 h before feeding at − 10, − 3, + 3, and + 17 d in milk (DIM). Blood gases, plasma metabolites and apparent total tract digestibility (ATTD) of nutrients were measured.

**Results:**

Feed intake, the ATTD of DM, energy, nitrogen, fat and crude fiber changed with DIM (*P* < 0.001). Except for Glu, O_2_, and urea, all net portal fluxes were positive, and all were affected by DIM (*P* < 0.05) and by sampling time (*P* < 0.01). Compared with pre partum levels, net portal uptake of AA was 3-63% lower at + 3 DIM but 40-100% higher at + 17 DIM. Net portal fluxes of AA peaked at 1.5 to 2.5 h after feeding except for Glu, and they were positively correlated with changes in sow feed intake across DIM. The net portal recovery was low for Met (49%), Thr (54%), and His (54%) and high for the remaining essential AA (63-69%) and none of them differed across DIM. Net hepatic uptake (i.e. hepatic oxidation) of Lys, Thr, Ile, Leu and Phe peaked at 0.5 to 2.5 h after feeding, whereas uptake of Trp, Val, and His was constant, while that of Met was close to zero.

**Conclusion:**

The net portal recovery was substantially lower for Met, Thr, and His than the remaining essential AA. Hepatic AA oxidation peaks 0.5 to 2.5 h after feeding. The heat production in PDV and liver was approximately two-fold higher at peak lactation compared to other stages. The study suggests that lysine was the limiting AA in peak lactation but not in early lactation.

## Introduction

In recent years, extensive research has been conducted to improve the efficiency of protein utilization and mitigate nitrogen losses to the environment [1–3]. These works have led to the development of diets with near optimal protein content and amino acid (AA) ratios in order to maximize reproductive performances of sows [4, 5]. However, it is challenging to estimate AA efficiencies from the diet because sows concomitantly mobilize AA from the body. Currently, it is not known whether the net portal uptake of AA changes across different physiological stages in response to substantial changes in feed intake, which would in turn affect the passage rate and net absorption profile [6]. During the transition and lactation period, many physiological processes related to reproductive output (fetal growth, mammary growth and milk production) are markedly changed [7], but knowledge on whether hepatic oxidation and net splanchnic release of AA change with the reproductive stage is lacking for the sow.

The portal-drained viscera (PDV; the intestine, pancreas, spleen and stomach) contributes approximately 35% of whole-body energy expenditure and protein synthesis in growing pigs, which plays an important role in the metabolism of both essential and non-essential AA [8]. Moreover, the liver, as a central organ regulating peripheral nutrient supply, is able to metabolize or catabolize AA [9]. Almost all the essential AA catabolic enzymes have been found in the liver, indicating that hepatic AA metabolism affects the supply of AA pattern to peripheral tissues. Therefore, quantifying the amount of AA being net absorbed from the gastrointestinal tract and net taken up and metabolized by the liver is important to understand the AA requirement during transition and lactation of sows. Meanwhile, quantitative knowledge about AA absorption and metabolism may contribute to maximize the efficiency of AA utilization in the future and to decrease nitrogen excretion and environmental pollution [10]. Energy loss due to heat production (HE) constitutes a substantial proportion of the metabolizable energy intake [11], but currently it is unknown how much of the total sow heat production may be ascribed to PDV and liver during transition and lactation [12].

To study the kinetics of AA metabolism in the PDV and in the liver, advanced surgical models are necessary. Surgical insertion of catheters combined with infusing an internal marker (*para*-aminohippuric acid, *p*AH) has been used to assess the plasma flow in e.g. the mammary vein in sows [13, 14] and in portal and hepatic veins of growing pigs [15]. In the present study, multiple catheters were surgically implanted to quantify the net portal and net hepatic AA metabolism in multiparous sows during late gestation and lactation to understand the post-prandial dynamics of net portal and net hepatic AA fluxes and to quantify the heat production of PDV and the liver.

## Materials and methods

The present experiment complied with the Danish Ministry of Justice Law 382 (June 10, 1987) and Act 726 (September 9, 1993, as amended by Act 1081 of December 20, 1995) concerning experiments with and the care of animals.

### Animals and surgical procedures

Eight second-parity sows (Danish Landrace × Yorkshire) were used in this study and housed in an intensive care facility at Research Center Foulum, Aarhus University, Denmark. On d 79 ± 3 of gestation, sows were surgically implanted with five permanent indwelling catheters in the right femoral artery, right femoral vein (not used in this study), the portal vein, the portal hepatic vein and the mesenteric vein as described by Kristensen et al. [15]. The mesenteric vein catheter was used for infusion of the blood flow marker *para*-amino hippuric acid (*p*AH). The *p*AH infusate contained 175 mmol/L of *p*AH and was adjusted to pH 7.4, sterile filtered (0.22 μm, FPE 214-500, JET Bio-Filtration Products Co., Ltd., Guangzhou, China), and autoclaved. At the end of the experiment, the positions of the catheters were investigated by autopsy to ensure correct placement.

### Feeding

Sows were fed a standard lactation diet containing wheat, barley and soybean meal from d − 15 relative to expected parturition and until weaning at d 28 of lactation. Chromic oxide (0.2%) was added to the diet as an indigestible marker for measuring the apparent total tract digestibility (ATTD) of nutrients. From 15 d prior to expected farrowing and until weaning, all sows were fed a lactation diet formulated to meet the Danish recommendations [16]. The dietary formulation is shown in Table 1. Feed was supplied three times daily in equal portions at 08:00, 16:00, and 24:00 h, and the restricted feed supply was registered, and feed leftovers were recorded and removed daily between 08:00 and 16:00 h, except on sampling days, when leftovers from the night meal were removed before the first sampling, and leftovers from the morning meal were removed 30 min after feeding. Feed intake corrected for refusals was recorded on a daily basis. The piglets had no access to creep feed, but both sows and piglets were offered water *ad libitum*.

**Table 1:** Dietary ingredients and chemical composition of the diet

Item	
Ingredients, (as fed)	g/kg
Barley	40.00
Wheat	35.62
Soybean meal, toasted	18.09
Animal fat	3.00
Monocalcium phosphate	1.19
Calcium carbonate	1.55
Sodium chloride	0.36
Vitamin-mineral premix	0.20
Cromium oxide	2.27
Chemical composition^1^	g/kg DM
DM, % (as fed)	91.18
Crude protein	193.64
Crude fat	55.44
Lysine	9.25 (7.87)
Methionine	2.88 (2.54)
Threonine	6.84 (5.64)
Tryptophan	2.35 (2.00)
Isoleucine	8.49 (7.28)
Leucine	14.39 (12.38)
Valine	9.95 (8.25)
Histidine	4.81 (4.16)
Phenylalanine	10.06 (8.73)
Alanine	8.13 (6.30)
Aspartate	16.45 (13.70)
Cysteine	3.59 (2.94)
Glutamate	44.11 (39.57)
Glycine	8.31 (6.57)
Proline	15.43 (11.96)
Serine	9.72 (8.23)
Tyrosine	5.58 (4.73)
Arginine	12.14 (10.90)

Composed to fulfill the recommendations for lactating sows by the Danish Pig Research Center (VSP, Copenhagen, Denmark)

^1^In parentheses: standardized ileal digestible (SID) content of individual AA (g/kg DM). The contents of SID Lys, Met, Thr, Ile, Leu, Val, His, Phe, and Cys were calculated based on feed ingredient compositions by DPRC (2013), and the SID contents of Trp, Ala, Asp, Glu, Gly, Pro, Ser, Tyr and Arg were calculated from feed ingredient compositions by NRC (2012)

### Samples and data collection

Sow body weight (BW) was measured on d − 10, − 3, + 3, + 17, and + 28 of lactation. Blood was sampled from the sows on d 105 ± 2 and 112 ± 2 of gestation (referred to as − 10 and − 3 d in milk [DIM]) and again during lactation at 3 ± 0 and 17 ± 2 DIM. On each sampling day, eight sets of blood samples were simultaneously drawn from the artery, the portal vein and the hepatic vein at hourly intervals from 0.5 h before to 6.5 h after feeding. Whole blood was collected for blood gas measurements, while plasma was obtained by centrifuging the blood sample at 1558×*g* at 4 °C for 12 min and stored at − 20 °C until analysis. Fecal samples on day − 10, − 3, + 3, and + 17 of lactation were collected to determine nutrient ATTD using chromic oxide as the external marker. The subsamples of diet were taken weekly and pooled for analysis.

### Chemical analysis

Fecal samples were freeze-dried and analyzed in duplicate for gross energy (GE), dry matter (DM), fat, nitrogen, crude fiber, and chromic oxide. Feed and fecal GE was determined using an adiabatic bomb calorimeter (Parr Instrument Company, Moline, Illinois, USA). The DM was determined by drying the samples at 105 °C to a constant weight, and crude fiber was analyzed according to the AOAC method (method 978.10, AOAC, 2007). The CP (N × 6.25) was determined by the Dumas method [17], and the fat after HCl hydrolysis was measured according to the Stoldt procedure [18]. Dietary AA was determined using an amino acid analyzer (Biochrom 20 plus; Biochrom Ltd., Cambridge, UK; [EC] 152/2009; European Commission 2009). Chromic oxide in diets and fecal samples was measured spectrophotometrically according to Schurch et al. [19].

Oxygen and carbon dioxide were measured in whole blood using an ABL700 blood gas analyzer (Radiometer, Copenhagen, Denmark). Plasma concentrations of deacetylated *p*AH and urea were measured as described by Larsen and Kristensen using a continuous flow analyzer (Autoanalyzer 3, method US-216-72 Rev. 1; Seal Analytical Ltd., Burgess Hill, UK) [20]. For the analysis of AA in plasma, proteins in 700-mL plasma samples were precipitated with 250 mL of a 32.4% sulphosalicylic acid solution. Then, the supernatant was applied to resin columns (AG 50 W-X8 Resin 100–200 mesh H^+^; Bio-Rad laboratories, Copenhagen, Denmark), washed twice with 2 mL of water, and the AA were subsequently eluted twice with 1 mL of 2 mol/L NH_4_OH followed by 1 mL of water. The AA eluate was analyzed for concentrations of Lys, Met, Thr, Trp, Ile, Leu, Val, Phe, His, Tyr, Ala, Asn, Asp, Gln, Glu, Gly, Pro, Ser, and Cys by gas chromatography–mass spectrometry (GCMS; Finigan Trace GC ultra-DSQ II; Thermo Scientific, Bremen, Germany) as described by Larsen et al. [21].

### Calculations and statistical analysis

Dietary crude protein content was calculated as N × 6.25. Nutrient ATTD was estimated using analyzed chromic oxide concentration in feed and feces according to Stein et al. [22] using the following equation (Eq.1):

1 $$ \mathrm{ATTD}=\left[1-\left(\frac{\mathrm{Nutrient}\ \mathrm{concentration}\ \mathrm{in}\ \mathrm{feces}}{\mathrm{Nutrient}\ \mathrm{concentration}\ \mathrm{in}\ \mathrm{diet}}\right)\times \left(\frac{\mathrm{Marker}\ \mathrm{concentration}\ \mathrm{in}\ \mathrm{diet}}{\mathrm{Marker}\ \mathrm{concentration}\ \mathrm{in}\ \mathrm{feces}}\right)\right]\times 100 $$

The portal plasma (Eq. 2) and blood flow (Eq. 3) were calculated according to the following equations:

2 $$ \mathrm{Portal}\ \mathrm{plasma}\ \mathrm{flows}\ \left(\mathrm{L}/\mathrm{h}\right)=\frac{\mathrm{infusion}\ \mathrm{rate}\ \mathrm{of}\ p\mathrm{AH}\ \left(\mathrm{mmol}/\mathrm{h}\right)}{\mathrm{portal}\ \mathrm{plasma}\ p\mathrm{AH}\ \mathrm{concentration}\ \left(\mathrm{mmol}/\mathrm{L}\right)-\mathrm{arterial}\ \mathrm{plasma}\ p\mathrm{AH}\ \mathrm{concentration}\ \left(\mathrm{mmol}/\mathrm{L}\right)} $$

3 $$ \mathrm{Portal}\ \mathrm{blood}\ \mathrm{flows}\ \left(\mathrm{L}/\mathrm{h}\right)=\frac{\mathrm{portal}\ \mathrm{plasma}\ \mathrm{flows}\ \left(\mathrm{L}/\mathrm{h}\right)}{1-\left(\mathrm{content}\ \mathrm{of}\ \mathrm{hematocrit}/100\right)} $$

The net portal flux (NPF) for O_2_, CO_2_, insulin, and urea was calculated according to the following equation (Eq. 4):

4 $$ \mathrm{NPF}\ \left(\mathrm{mmol}/\mathrm{h}\right)=\mathrm{portal}\ \mathrm{blood}\ \mathrm{or}\ \mathrm{plasma}\ \mathrm{flow}\ \left(\mathrm{L}/\mathrm{h}\right)\times \left[\mathrm{portal}\ \mathrm{concentration}\ \left(\mathrm{mmol}/\mathrm{L}\right)-\mathrm{arterial}\ \mathrm{concentration}\ \left(\mathrm{mmol}/\mathrm{L}\right)\right] $$

The NPF of AA (in g/d) was calculated according to the equation (Eq. 5):

5 $$ \mathrm{NPF}\ \left(\mathrm{g}/\mathrm{d}\right)=\mathrm{NPF}\ \left(\mathrm{mmol}/\mathrm{h}\right)\times 24\ \left(\mathrm{h}/\mathrm{d}\right)\times \mathrm{molar}\ \mathrm{mass}\ \mathrm{for}\ \mathrm{each}\ \mathrm{AA}\ \left(\mathrm{g}/\mathrm{mol}\right)\times 0.001\ \left(\mathrm{mol}/\mathrm{mmol}\right) $$

The hepatic plasma (Eq. 6) and blood flow (Eq. 7) were calculated according to the following equations:

6 $$ \mathrm{Hepatic}\ \mathrm{plasma}\ \mathrm{flow}\ \left(\mathrm{L}/\mathrm{h}\right)=\frac{\mathrm{infusion}\ \mathrm{rate}\ \mathrm{of}\ p\mathrm{AH}\ \left(\mathrm{mmol}/\mathrm{h}\right)}{\ \mathrm{hepatic}\ p\mathrm{AH}\ \mathrm{concentration}\ \left(\mathrm{mmol}/\mathrm{L}\right)-\mathrm{arterial}\ \mathrm{plasma}\ p\mathrm{AH}\ \mathrm{concentration}\ \left(\mathrm{mmol}/\mathrm{L}\right)} $$

7 $$ \mathrm{Hepatic}\ \mathrm{blood}\ \mathrm{flow}\ \left(\mathrm{L}/\mathrm{h}\right)=\frac{\mathrm{hepatic}\ \mathrm{plasma}\ \mathrm{flow}\mathrm{s}\ \left(\mathrm{L}/\mathrm{h}\right)}{1-\left(\mathrm{content}\ \mathrm{of}\ \mathrm{hematocrit}/100\right)} $$

The net hepatic flux (NHF) for O_2_, CO_2_, insulin, and urea was calculated according to the following equation (Eq. 8):

8 $$ \mathrm{NHF}\ \left(\mathrm{mmol}/L\right)=\mathrm{hepatic}\ \mathrm{blood}\ \mathrm{or}\ \mathrm{plasma}\ \mathrm{flow}\ \left(\mathrm{L}/\mathrm{h}\right)\times \mathrm{hepatic}\ \mathrm{concentration}\ \left(\mathrm{mmol}/\mathrm{L}\right)-\left\{\mathrm{portal}\ \mathrm{blood}\ \mathrm{or}\ \mathrm{plasma}\ \mathrm{flow}\ \left(\mathrm{L}/\mathrm{h}\right)\times \mathrm{portal}\ \mathrm{concentration}\ \left(\mathrm{mmol}/\mathrm{L}\right)+\left[\mathrm{hepatic}\ \mathrm{blood}\ \mathrm{or}\ \mathrm{plasma}\ \mathrm{flow}\ \left(\mathrm{L}/\mathrm{h}\right)-\mathrm{portal}\ \mathrm{blood}\ \mathrm{or}\ \mathrm{plasma}\ \mathrm{flow}\ \left(\mathrm{L}/\mathrm{h}\right)\right]\times \mathrm{arterial}\ \mathrm{concentration}\ \left(\mathrm{mmol}/\mathrm{L}\right)\right\} $$

The NHF for AA (in g/d) was calculated according to the equation (Eq. 9):

9 $$ \mathrm{NHF}\ \left(\mathrm{g}/\mathrm{d}\right)=\mathrm{NHF}\ \left(\mathrm{mmol}/\mathrm{h}\right)\times 24\ \left(\mathrm{h}/\mathrm{d}\right)\times \mathrm{molar}\ \mathrm{mass}\ \mathrm{for}\ \mathrm{each}\ \mathrm{AA}\ \left(\mathrm{g}/\mathrm{mol}\right)\times 0.001\ \left(\mathrm{mol}/\mathrm{mmol}\right) $$

Daily individual AA intake was calculated by multiplying the feed intake with the content of AA in the diet as follows (Eq. 10). The net portal recovery (Eq. 11) and hepatic extraction (Eq. 12) of AA were calculated according to the following equations:

10 $$ \mathrm{AA}\ \mathrm{intake}\ \left(\mathrm{g}/\mathrm{d}\right)=\mathrm{Feed}\ \mathrm{intake}\ \left(\mathrm{kg}/\mathrm{d}\right)\times \mathrm{DM}\ \left(\%\right)/100\times \mathrm{content}\ \mathrm{of}\ \mathrm{AA}\ \left(\mathrm{g}/\mathrm{kg}\right) $$

11 $$ \mathrm{Net}\ \mathrm{portal}\ \mathrm{recovery}\ \left(\%\right)=\frac{\mathrm{NPF}\ \left(\mathrm{g}/\mathrm{d}\right)\ }{\mathrm{AA}\ \mathrm{intake}\ \left(\mathrm{g}/\mathrm{d}\right)} \times 100 $$

12 $$ \mathrm{Hepatic}\ \mathrm{extraction}\ \left(\%\right)=\frac{-\mathrm{NHF}\ \left(\mathrm{mmol}/\mathrm{L}\right)}{\mathrm{Portal}\ \mathrm{flow}\ \left(\mathrm{L}/\mathrm{h}\right)\times \mathrm{portal}\ \mathrm{concentration}\ \mathrm{of}\ \mathrm{AA}\ \left(\mathrm{mmol}/\mathrm{L}\right)+\left[\mathrm{Hepatic}\ \mathrm{flow}\ \left(\mathrm{L}/\mathrm{h}\right)-\mathrm{Portal}\ \mathrm{flow}\ \left(\mathrm{L}/\mathrm{h}\right)\right]\times \mathrm{arterial}\ \mathrm{concentration}\ \mathrm{of}\ \mathrm{AA}\ \left(\mathrm{mmol}/\mathrm{L}\right)} \times 100 $$

The net splanchnic recovery was calculated according to the following equation (Eq. 13):

13 $$ \mathrm{Net}\ \mathrm{splanchnic}\ \mathrm{recovery}\ \left(\%\right)=\frac{\mathrm{hepatic}\ \mathrm{plasma}\ \mathrm{flow}\ \left(\mathrm{L}/\mathrm{h}\right)\times \left[\mathrm{hepatic}\ \mathrm{AA}\ \mathrm{concentration}\ \left(\mathrm{mmol}/\mathrm{L}\right)-\mathrm{arterial}\ \mathrm{AA}\ \mathrm{concentration}\ \left(\mathrm{mmol}/\mathrm{L}\right)\right]\times 24\ \left(\mathrm{h}/\mathrm{d}\right)\times \mathrm{molar}\ \mathrm{mass}\ \mathrm{for}\ \mathrm{each}\ \mathrm{AA}\ \left(\mathrm{g}/\mathrm{mol}\right)/1000\ }{\mathrm{AA}\ \mathrm{intake}\ \left(\mathrm{g}/\mathrm{d}\right)} \times 100 $$

Positive net fluxes indicate net absorption from the GI-tract or net synthesis (or net release) from the liver, whereas negative net fluxes indicate net uptake, metabolism or catabolism of the substance. Heat production in PDV and liver was calculated with the following formula adapted from Brouwer [23]: HE = 16.18 × VO_2_ consumed (L/d) + 5.02 × VCO_2_ produced (L/d); VO_2_ and VCO_2_ are the volumes (L) of O_2_ and CO_2_, respectively. Volumes of O_2_ and CO_2_ were converted from net fluxes of these blood gases (in mmol/h) into L/d using the gas equation: V = n × R × T/P, where V = volume, n = number of moles per day, R = gas constant, T = temperature, P = pressure. Prior to farrowing, total HE was calculated according to Feyera and Theil [7]. After farrowing, total HE was estimated by assuming that lactating sows require 0.482 MJ/(kg^0.75^ × d) to maintenance, and heat energy due to milk production was estimated by assuming a *k*_l_ of 0.78 [11]. The respiratory quotient (RQ) was determined as CO_2_ produced/O_2_ consumed ratio.

All statistical analyses were performed using the MIXED procedure of SAS version 9.3 (SAS Inst. Inc., Cary, NC). The model included fixed effects of DIM (− 10, − 3, 3, 17), sampling time relative to feeding (− 0.5, 1.5, 2.5, 3.5, 4.5, 5.5, 6.5 h), and the two-way interaction between fixed effects. The net flux data were whether they differed statistically from zero, and this information was included in the respective Tables. The sow was included as a random effect using a compound symmetry covariance structure to account for repeated measurements within sows across DIM. To account for repeated measurements within a sampling day, a first order autoregressive covariance structure was applied using a repeated statement. The Tukey-Kramer test was used in multiple comparisons of means to adjust the *P*-values. Variables with only one observation per day were analyzed using a reduced model including the fixed effect of DIM. Correlation analysis between insulin and NPF or NHF of AA was analyzed by CORR procedure of SAS and results are represented by Pearson correlations (*r*) and level of significance (*P*). Data were presented as means ± SEM. A probability value of *P* < 0.05 was considered as statistically significant.

## Results

### Feed intake, sows body weight and digestibility

The feed intake was intermediate pre partum, lowest at + 3 DIM (20% lower than pre partum), and highest at + 17 DIM (95% higher than pre partum feed intake; Table 2). In contrast, the digestibility of DM, GE, nitrogen, fat, and crude fiber was highest at + 3 DIM, intermediate pre partum, and lowest at + 17 DIM (*P* < 0.01). Sow BW decreased (*P* < 0.001) from late gestation to early and peak lactation.

**Table 2:** Sow performance, feed intake, and digestibility of sows during late gestation and in early and peak lactation

Item	DIM^1^				SEM	*P*-value
	−10	−3	3	17		
Feed intake, kg/d	3.32^b^	3.34^b^	2.66^c^	6.51^a^	0.12	< 0.001
Sow BW^1^, kg	262^a^	271^a^	244^b^	234^b^	52	< 0.001
Digestibility, g/100 g						
DM^1^	83.3^b^	83.3^b^	86.2^a^	79.8^c^	0.7	< 0.001
GE^1^	82.5^b^	82.8^b^	86.1^a^	79.1^c^	0.8	< 0.001
Nitrogen	83.4^b^	84.2^b^	89.0^a^	80.3^c^	0.8	< 0.001
Fat	61.8^a^	63.7^a^	66.4^a^	53.2^b^	1.8	< 0.001
Crude Fiber	68.7^ab^	66.4^bc^	74.8^a^	61.4^c^	2.2	0.003

^a–c^Within a row and within a main effect (DIM), means with different letters differ

^1^*DIM* days in milk; *BW* body weight; *DM* dry matter; *GE* gross energy

### Arterial plasma concentration

**Fig. 1:**
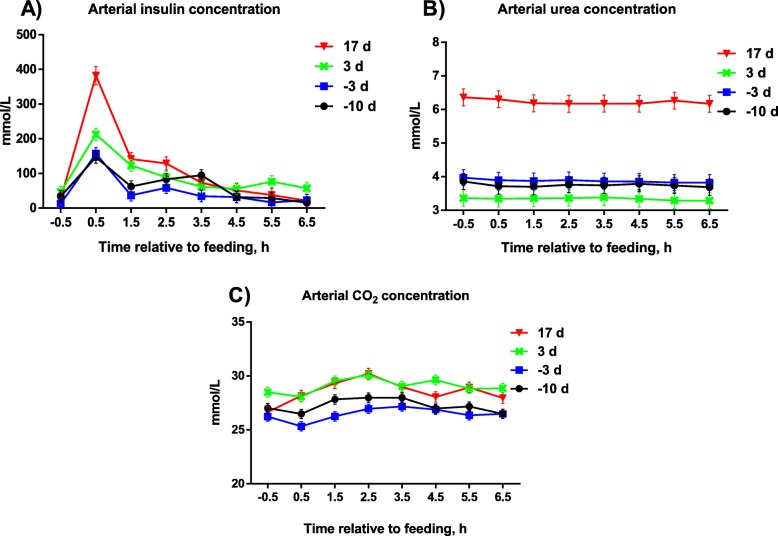
Arterial insulin (**a**), urea (**b**), and CO_2_ (**c**) concentrations of sows during late gestation and in early and peak lactation. Influences of DIM were observed for arterial insulin (*P* < 0.001), urea (*P* < 0.001), and CO_2_ (*P* < 0.001) concentrations, respectively. Influences of time after feeding were observed for arterial insulin (*P* < 0.001), urea (*P* < 0.001), and CO_2_ (*P* < 0.001) concentrations, respectively. Interactions between time after feeding and DIM were observed for arterial insulin (*P* < 0.001), urea (*P* = 0.02), and CO_2_ (*P* = 0.008) concentrations, respectively. DIM, days in milk

All measured arterial concentrations of metabolites, except O_2_, Met, Thr, His, and Gly, were affected by DIM (*P* < 0.05; Table 3 and Fig. 1). All measured arterial concentrations of metabolites were affected by ST relative to feeding (*P* < 0.001). Interactions between DIM and ST relative to feeding were observed for CO_2_ (*P* = 0.008), insulin (*P* < 0.001), urea (*P* = 0.02), Ala (*P* = 0.04), Asn (*P* = 0.03), Gly (*P* < 0.001), and Ser (*P* = 0.01). Plasma CO_2_ and plasma insulin were greatest at + 3 and + 17 DIM, and plasma urea was greatest at + 17 DIM. Plasma Lys was greatest at + 3 DIM and lowest at + 17 DIM. Plasma Trp, Ile, Leu, Val, and Phe were greatest pre partum and lowest at + 3 DIM as was the case for the following non-essential AA: Ala, Asn, Pro, Ser, and Tyr. Arterial concentration of Gly peaked at 3.5 h after feeding, whereas arterial concentrations of all other AA peaked at 0.5-2.5 h after feeding.

**Table 3:** Hematocrit and arterial concentration of gases, insulin, urea, and amino acids in sows during late gestation and in early and peak lactation

Item	DIM^1^				SEM	ST^1^								SEM	*P*-value		
	-10	-3	3	17		−0.5	0.5	1.5	2.5	3.5	4.5	5.5	6.5		DIM	ST	ST×DIM
Hematocrit, %	27.5^ab^	28.1^a^	27.0^bc^	26.3^c^	0.5	28.4^a^	28.4^a^	26.8^b^	26.6^b^	26.7^b^	26.8^b^	26.9^b^	27.1^b^	0.5	0.008	< 0.001	0.54
O_2_, mmol/L	5.9	5.8	5.7	5.8	0.1	6.2^a^	6.1^a^	5.8^b^	5.7^b^	5.6^b^	5.6^b^	5.7^b^	5.7^b^	0.1	0.46	< 0.001	0.64
CO_2_, mmol/L	27.2^b^	26.5^c^	29.1^a^	28.5^a^	0.3	27.1^d^	27.0^d^	28.2^b^	28.8^a^	28.3^b^	27.9^bc^	27.8^bc^	27.4^cd^	0.3	< 0.001	< 0.001	0.008
Insulin, mmol/L	62.2^b^	46.3^b^	90.3^a^	107.9^a^	7.7	29.9^d^	224.7^a^	90.9^b^	90.0^b^	66.1^c^	42.6^d^	39.9^d^	29.3^d^	10.1	< 0.001	< 0.001	< 0.001
Urea, mmol/L	3.7^b^	3.9^b^	3.3^c^	6.2^a^	0.2	4.4^a^	4.3^b^	4.3^c^	4.3^bc^	4.3^bc^	4.3^bc^	4.3^c^	4.2^d^	0.2	< 0.001	< 0.001	0.02
Essential AA, μmol/L																	
Lys	97.0^b^	90.3^b^	133.9^a^	84.5^b^	12.0	86.1^d^	114.1^ab^	112.1^ab^	116.7^a^	108.3^b^	99.7^c^	88.4^d^	85.9^d^	8.4	0.005	< 0.001	0.77
Met	28.3	27.1	26.0	24.9	1.8	25.6^d^	31.3^a^	28.5^b^	26.7^cd^	27.5^bc^	25.6^d^	23.7^e^	24.0^de^	1.4	0.37	< 0.001	0.68
Thr	174.3	189.6	180.7	163.3	14.1	163.4^d^	185.5^ab^	186.3^ab^	190.0^a^	184.0^b^	177.8^c^	166.7^d^	162.1^d^	11.9	0.17	< 0.001	0.55
Trp	69.3^a^	68.5^a^	56.1^b^	66.2^a^	3.7	61.6^d^	67.4^ab^	68.0^ab^	69.2^a^	66.5^bc^	64.8^c^	61.9^d^	60.8^d^	3.5	< 0.001	< 0.001	0.38
Ile	166.2^a^	158.5^a^	108.2^b^	118.7^b^	6.2	126.5^c^	147.0^a^	143.3^ab^	144.4^ab^	141.0^ab^	139.2^b^	131.1^c^	130.5^c^	5.1	< 0.001	< 0.001	0.88
Leu	205.4^a^	182.6^b^	139.7^c^	151.2^c^	10.0	154.7^c^	188.3^a^	178.4^b^	176.5^b^	171.2^b^	169.4^b^	159.8^c^	159.3^c^	8.8	< 0.001	< 0.001	0.44
Val	340.2^a^	290.3^b^	266.5^b^	266.4^b^	14.5	271.4^c^	307.5^a^	304.1^ab^	301.7^ab^	295.5^ab^	292.0^b^	278.9^c^	275.7^c^	11.3	< 0.001	< 0.001	0.51
His	82.2	81.5	82.7	89.4	4.3	77.6^c^	85.0^ab^	87.7^a^	88.3^a^	87.4^a^	86.3^a^	80.9^bc^	78.2^c^	4.0	0.09	< 0.001	0.71
Phe	107.2^a^	103.6^a^	84.3^b^	102.8^a^	5.4	88.3^d^	102.9^ab^	105.2^ab^	106.8^a^	103.8^ab^	101.9^b^	94.2^c^	92.8^cd^	5.1	< 0.001	< 0.001	0.82
Non-essential AA, μmol/L																	
Ala	545.8^a^	562.5^a^	478.4^b^	473.1^b^	28.7	454.5^d^	545.1^b^	580.8^a^	548.0^b^	531.7^bc^	515.2^c^	474.3^d^	469.9^d^	27.1	< 0.001	< 0.001	0.04
Asn	23.9^a^	20.9^b^	10.1^c^	21.1^b^	1.8	12.1^f^	18.0^d^	21.9^bc^	25.0^a^	22.7^b^	20.6^c^	16.6^de^	15.0^e^	1.7	< 0.001	< 0.001	0.03
Asp	41.8^b^	40.0^b^	46.7^a^	49.1^a^	2.1	36.7^e^	43.8^cd^	47.3^b^	51.4^a^	48.8^b^	46.7^bc^	42.0^d^	38.4^e^	1.8	0.001	< 0.001	0.57
Cys	205.9^b^	204.6^b^	260.5^a^	200.8^b^	7.9	223.0^a^	225.6^a^	215.1^b^	215.2^b^	216.2^b^	214.7^b^	216.4^b^	217.5^b^	6.1	< 0.001	< 0.001	0.33
Glu	254.1^ab^	234.6^bc^	269.3^a^	225.5^c^	13.5	230.3^c^	250.6^ab^	255.7^a^	253.5^a^	251.0^a^	249.3^ab^	239.7^bc^	236.7^c^	10.5	0.01	< 0.001	0.09
Gly	821.7	855.2	778.2	781.5	30.0	793.6d^e^	799.2^de^	780.3^e^	822.2^bc^	838.0^a^	828.1^ab^	810.1^cd^	801.7^cde^	16.9	0.16	< 0.001	< 0.001
Pro	501.4^a^	475.5^a^	320.3^c^	416.4^b^	26.5	371.4^e^	417.5^cd^	444.1^ab^	460.0^a^	455.6^a^	450.1^a^	427.5^bc^	401.0^de^	21.3	< 0.001	< 0.001	0.45
Ser	147.7^a^	142.1^a^	125.2^b^	140.8^a^	6.2	130.4^c^	146.1^a^	139.0^bc^	144.6^a^	143.3^ab^	140.8^ab^	134.6^c^	132.9^c^	4.8	0.010	< 0.001	0.01
Tyr	120.6^ab^	115.0^b^	89.2^c^	124.7^a^	5.8	103.3^d^	117.4^ab^	117.7^ab^	120.1^a^	116.5^b^	113.8^b^	107.0^c^	103.4^d^	5.3	< 0.001	< 0.001	0.44
Gln	276.2^c^	241.5^d^	344.2^b^	374.3^a^	14.1	290.0^c^	308.6^b^	318.0^b^	339.2^a^	338.1^a^	317.0^b^	290.2^c^	271.4^d^	13.1	< 0.001	< 0.001	0.33

^a–f^Within a row and within a main effect (DIM, ST), means with different letters differ

^1^*DIM* days in milk, *ST* sampling time

### Portal plasma and blood flows, net portal fluxes of O_2_, CO_2_ and amino acids

**Fig. 2:**
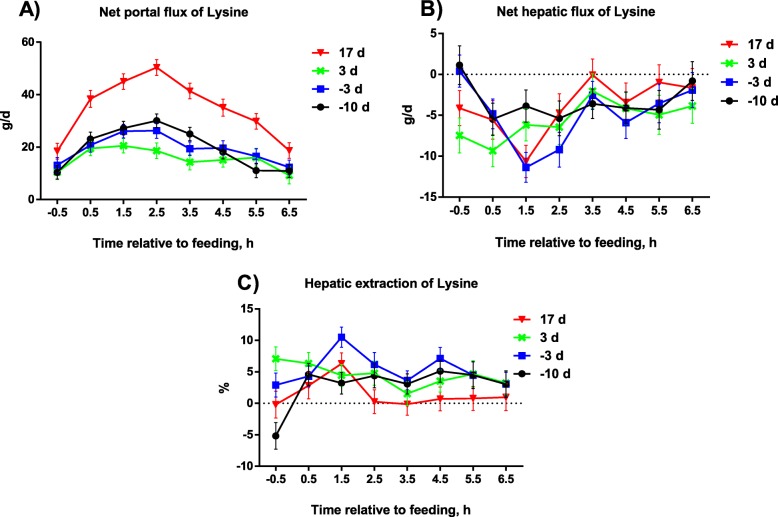
Net portal flux (**a**), net hepatic flux (**b**), and hepatic extraction (**c**) of lysine in sows during late gestation and in early and peak lactation. Influences of DIM were observed for net portal (*P* < 0.001), net hepatic (*P* = 0.12) fluxes, and hepatic extraction (*P* < 0.001) of lysine, respectively. Influences of time after feeding were observed for net portal (*P* < 0.001), net hepatic (*P* < 0.001) fluxes, and hepatic extraction (*P* = 0.008) of lysine, respectively. Interactions between time after feeding and DIM were observed for net portal (*P* = 0.02), net hepatic (*P* = 0.09), and hepatic extraction (*P* = 0.09) fluxes of lysine, respectively. DIM, days in milk

The net portal O_2_ flux was affected by DIM (*P* < 0.001) but not influenced by ST (*P* > 0.05; Table 4). All other net portal fluxes as well as plasma and blood flows were affected both by DIM (*P* < 0.05) and by ST (*P* < 0.01). Interactions between DIM and ST relative to feeding were observed for Lys (*P* = 0.02; Fig. 2), Leu (*P* = 0.008), His (*P* = 0.01), Phe (*P* = 0.003), Ala (*P* = 0.02), Asn (*P* = 0.003), Asp (*P* = 0.009), Pro (*P* = 0.006), and Tyr (*P* = 0.01). The net portal fluxes of these AA in + 17 DIM were greater than in other periods from 0.5-5.5 h after feeding. Compared with pre partum levels, portal plasma and blood flow and O_2_ and CO_2_ fluxes were similar at + 3 DIM while they were higher at + 17 DIM. Net insulin secretion and net urea release to the GI tract during lactation were higher than pre partum levels. Net portal uptake of all AA were lower at + 3 DIM and higher at + 17 DIM as compared with pre partum levels. However, in contrast to all other AA, net portal flux of Glu was negative most pronounced pre partum and at + 3 DIM, whereas at + 17 DIM it was essentially zero. Relative to feeding, the plasma and blood flow and CO_2_ flux peaked at 0.5-1.5 h after feeding, while net flux of all AA except for Glu peaked at 1.5-2.5 h after feeding.

**Table 4:** Portal plasma and blood flow, and net portal fluxes of gases, insulin, urea, and amino acids in sows during late gestation and in early and peak lactation^1^

Item	DIM^2^				SEM	ST^2^								SEM	*P*-value		
	−10	−3	3	17		−0.5	0.5	1.5	2.5	3.5	4.5	5.5	6.5		DIM	ST	ST×DIM
Portal flows, L/h																	
Plasma	164^b^	164^b^	167^b^	274^a^	7	189^b^	205^a^	208^a^	201^ab^	195^ab^	188^b^	187^b^	168^c^	7	< 0.001	< 0.001	0.46
Blood	227^b^	228^b^	229^b^	375^a^	9	262^c^	294^a^	282^ab^	273^bc^	265^bc^	256^c^	254^c^	230^d^	10	< 0.001	< 0.001	0.71
Net portal fluxes																	
O_2_, mmol/h	− 457^b^	− 441^b^	− 411^b^	− 718^a^	22	− 479	− 522	− 555	− 546	− 506	− 503	− 492	− 451	26	< 0.001	0.07	0.06
CO_2_, mmol/h	560^b^	534^b^	503^b^	936^a^	28	639^ab^	701^a^	685^ab^	658^ab^	599^b^	652^ab^	619^ab^	509^c^	36	< 0.001	0.007	0.07
Insulin, mol/h	23.5^b^	17.9^b^	32.3^a^	35.7^a^	2.7	15.6^de^	70.8^a^	28.5^bc^	32.9^b^	22.9^cd^	20.6^cd^	16.3^de^	10.9^e^	3.4	< 0.001	< 0.001	0.002
Urea, mmol/h	−5.0^b^	(−2.6^b^)	−6.2^b^	−21.1^a^	1.8	(−4.0)	−11.2	−9.9	−7.7	−8.7	−6.9	−10.6	−10.6	2.6	< 0.001	0.72	0.01
Essential AA, g/d																	
Lys	19.5^b^	19.3^b^	15.5^c^	34.7^a^	1.4	13.1^d^	25.4^b^	29.7^a^	31.3^a^	25.0^b^	22.0^b^	18.4^c^	12.8^d^	1.6	< 0.001	< 0.001	0.02
Met	4.2^b^	3.8^b^	3.7^b^	8.0^a^	0.6	1.5^c^	7.2^a^	7.1^a^	6.8^a^	5.7^ab^	4.6^b^	4.3^b^	2.1^c^	0.6	< 0.001	< 0.001	0.95
Thr	11.8^b^	11.8^b^	8.0^c^	21.3^a^	0.9	7.2^e^	15.2^bc^	17.7^ab^	18.9^a^	15.7^b^	12.7^cd^	10.8^d^	7.5^e^	1.1	< 0.001	< 0.001	0.10
Trp	4.8^b^	4.7^b^	3.9^b^	8.9^a^	0.4	2.5^d^	6.0^bc^	7.6^a^	7.5^a^	6.8^ab^	5.9^bc^	5.0^c^	3.1^d^	0.5	< 0.001	< 0.001	0.22
Ile	17.0^b^	16.1^b^	13.1^c^	30.9^a^	1.1	10.5^d^	20.0^bc^	26.2^a^	27.0^a^	21.3^b^	20.1^bc^	17.0^c^	12.2^d^	1.4	< 0.001	< 0.001	0.25
Leu	29.5^b^	28.2^b^	22.6^c^	52.1^a^	1.7	16.8^d^	38.2^b^	44.3^a^	45.5^a^	36.8^b^	33.6^bc^	29.3^c^	20.6^d^	2.1	< 0.001	< 0.001	0.008
Val	19.1^b^	19.5^b^	14.5^c^	34.4^a^	1.5	13.0^d^	21.9^bc^	28.7^a^	30.5^a^	26.2^ab^	22.2^bc^	18.3^c^	14.2^cd^	1.8	< 0.001	< 0.001	0.25
His	8.9^b^	7.6^b^	5.1^c^	16.0^a^	0.9	5.8^cd^	11.6^b^	12.2^ab^	14.8^a^	12.9^ab^	7.9^c^	4.4^d^	5.7^cd^	1.0	< 0.001	< 0.001	0.01
Phe	21.6^b^	20.8^b^	15.9^c^	38.7^a^	1.2	13.2^e^	27.4^bc^	31.9^a^	32.1^a^	28.9^ab^	24.2^cd^	21.8^d^	14.6^e^	1.5	< 0.001	< 0.001	0.003
Non-essential AA, g/d																	
Ala	50.8^b^	44.9^b^	37.3^c^	93.0^a^	2.7	35.7^e^	66.7^ab^	73.9^a^	71.1^ab^	59.9^bc^	57.2^cd^	49.4^d^	38.0^e^	3.3	< 0.001	< 0.001	0.02
Asn	9.8^b^	8.7^b^	5.5^c^	14.7^a^	0.6	4.4^e^	9.7^c^	14.2^a^	14.1^a^	11.7^b^	9.8^c^	7.8^d^	5.7^e^	0.7	< 0.001	< 0.001	0.003
Asp	8.8^b^	8.7^b^	8.5^b^	14.0^a^	0.6	6.0^d^	11.0^b^	12.8^a^	13.6^a^	11.0^b^	10.6^b^	8.3^c^	6.6^d^	0.7	< 0.001	< 0.001	0.009
Cys	5.4^b^	5.9^b^	2.7^c^	9.0^a^	0.6	4.4^cd^	5.2^bc^	6.6^ab^	8.6^a^	5.7^bc^	7.1^ab^	5.9^b^	2.7^d^	0.8	< 0.001	< 0.001	0.18
Glu	−5.3^a^	−4.5^a^	−4.2^a^	(−0.6^b^)	1.0	−7.6^a^	−4.1^b^	−2.8^b^	(−2.2^bc^)	(0.5^c^)	−3.9^b^	− 3.9^b^	−5.3^ab^	1.2	0.003	< 0.001	0.75
Gly	34.6^b^	32.6^b^	30.9^b^	63.5^a^	2.3	38.6^bcd^	37.0^cd^	42.4^abc^	43.4^ab^	40.1^abcd^	44.9^a^	41.9^abc^	34.8^d^	2.7	< 0.001	0.01	0.42
Pro	34.2^b^	32.0^b^	24.0^c^	64.6^a^	2.7	20.3^e^	42.3^b^	52.9^a^	51.6^a^	42.5^b^	41.0^bc^	33.6^c^	25.3^d^	3.2	< 0.001	< 0.001	0.006
Ser	19.1^b^	18.1^b^	13.8^c^	34.0^a^	1.2	10.6^d^	24.3^b^	27.8^a^	29.3^a^	24.6^ab^	21.9^b^	18.2^c^	13.1^d^	1.4	< 0.001	< 0.001	0.15
Tyr	15.5^b^	14.7^b^	11.3^c^	27.2^a^	0.9	8.3^d^	20.0^b^	22.8^a^	23.7^a^	20.1^b^	17.9^b^	14.4^c^	10.1^d^	1.0	< 0.001	< 0.001	0.01
Gln	12.4^a^	12.2^a^	(4.6^b^)	17.2^a^	2.9	(1.1^c^)	12.2^b^	21.7^a^	18.2^ab^	14.9^ab^	15.6^ab^	7.5^c^	(1.4)	3.7	0.03	< 0.001	0.85

^a–e^Within a row and within a main effect (DIM, ST), means with different letters differ. ^1^Values presented in parenthesis are not significantly different from zero. ^2^*DIM*, days in milk, *ST* sampling time

### Net portal recovery

Net portal recoveries of essential AA did not differ across DIM (Table 5; *P* > 0.05) and were all within the range of 45-72%. The net portal recovery of Asp, Cys and Glu were affected markedly by DIM (*P* < 0.05), while the remaining non-essential AA did not differ across DIM (*P* > 0.05). The net portal recovery rate of non-essential AA varied much more than the essential AA, ranging from − 4% (Glu) to 208% (Ala).

**Table 5:** Net portal recovery of amino acids in sows during late gestation and in early and peak lactation

Item	DIM^1^				SEM	*P*-value
	−10	−3	3	17		
Essential AA, %						
Lys	70.5	69.8	70.0	63.7	4.8	0.70
Met	48.8	45.7	54.9	45.1	6.6	0.66
Thr	57.4	58.4	48.9	52.6	4.4	0.31
Trp	67.8	67.1	69.8	64.0	4.2	0.79
Ile	66.8	63.6	65.8	61.3	3.8	0.70
Leu	68.3	65.7	66.8	61.6	3.4	0.51
Val	64.1	65.4	62.3	59.0	4.5	0.73
His	62.0	54.6	46.9	54.4	5.4	0.21
Phe	72.1	69.3	67.3	65.3	3.8	0.57
Nonessential AA, %						
Ala	207.6	184.7	192.0	191.3	9.9	0.31
Asp	17.7^b^	17.6^b^	21.3^a^	14.6^c^	1.1	0.002
Cys	49.6^a^	55.1^a^	32.6^b^	42.6^ab^	5.9	0.04
Glu	−4.0^a^	−3.3^a^	− 3.9^a^	−0.3^b^	0.7	< 0.001
Gly	137.3	129.2	152.8	129.7	8.7	0.16
Pro	73.6	69.3	66.3	69.0	5.9	0.80
Ser	65.6	62.9	59.4	58.9	4.4	0.61
Tyr	92.2	89.1	85.3	82.5	5.0	0.48

^a–c^Within a row and within a main effect (DIM), means with different letters differ

^1^*DIM* days in milk

### Hepatic plasma and blood flows, net hepatic fluxes of O_2_, CO_2_ and amino acids

**Fig. 3:**
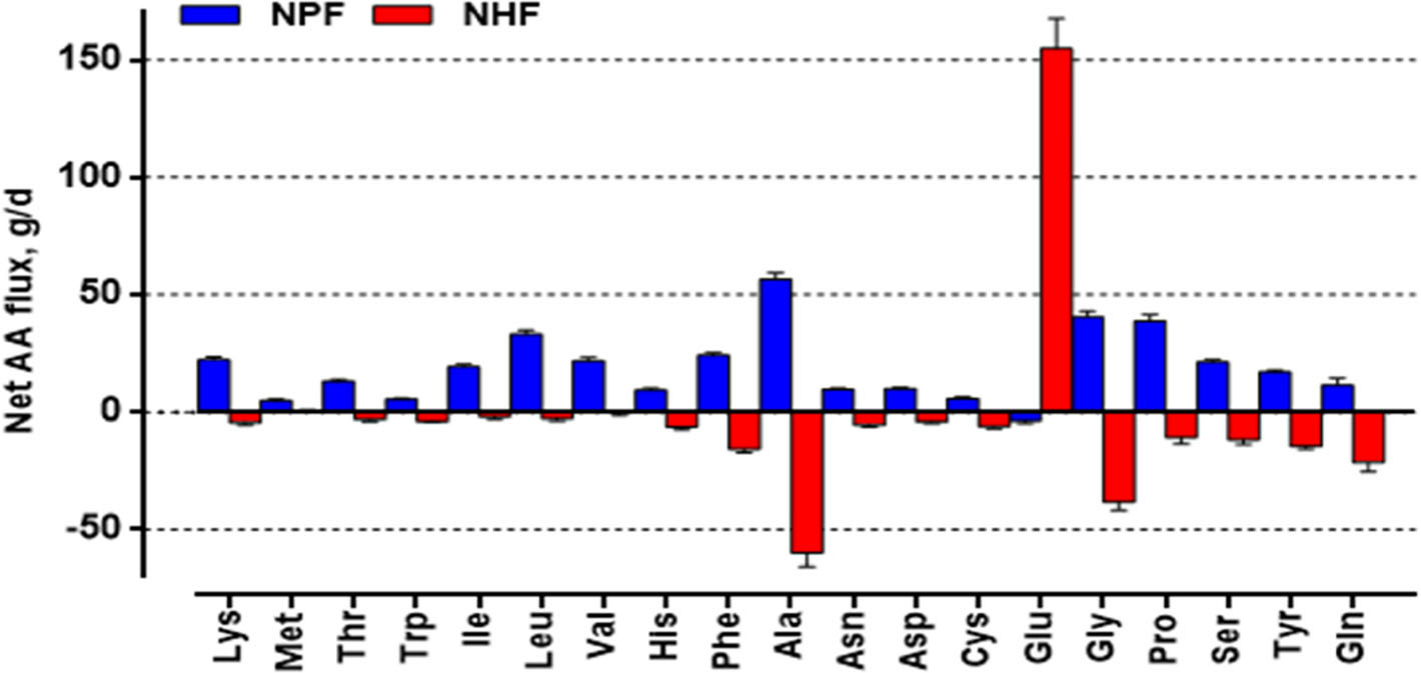
The average net portal fluxes (NPF, blue bars) and net hepatic fluxes (NHF, red bars) of amino acids in sows during late gestation and lactation

The hepatic plasma flow, blood flow, and net hepatic flux of CO_2_ were affected by DIM (*P* < 0.001) and ST (*P* < 0.01), respectively (Table 6). The net hepatic O_2_ flux was affected by DIM (*P* < 0.001) but not influenced by ST (*P* > 0.05). The net hepatic flux of most AA were negative (Fig. 3), but Met was close to zero and net hepatic flux of Glu was highly positive. The net hepatic fluxes of Trp, His, Phe, Ala, Asn, Asp, Glu, Gly, Pro, Ser, Tyr and Gln were affected by DIM (*P* < 0.05), while the net hepatic fluxes of Lys, Thr, Ile, Leu, Phe, Ala, Asn, Asp, Cys, Glu, Ser and Tyr were influenced by ST relative to feeding (*P* < 0.05). In addition, interactions between DIM and ST relative to feeding were observed for Leu (*P* = 0.006), Ala (*P* < 0.001), Asn (*P* = 0.02), Gly (*P* = 0.002) and Ser (*P* = 0.001). Compared to pre partum levels, hepatic plasma and blood, O_2_, CO_2_, urea, Trp, His, Ala, Asn, Asp, Gly, and Tyr fluxes were similar at + 3 DIM, while they were higher at + 17 DIM. Net hepatic insulin secretion and hepatic fluxes of Glu and Gln in lactation were higher than the pre partum levels. Net hepatic fluxes of Phe, Pro, and Ser at + 3 DIM were lower than the other period. Relative to feeding, the plasma and blood flow, O_2_, CO_2_, and all AA fluxes peaked at 0.5-2.5 h after feeding, except for Val and His.

**Table 6:** Hepatic blood and plasma flow, and net hepatic fluxes of gases, insulin, urea, and amino acids in sows during late gestation and in early and peak lactation^**1**^

Item	DIM^2^				SEM	ST^2^								SEM	*P*-value		
	−10	−3	3	17		− 0.5	0.5	1.5	2.5	3.5	4.5	5.5	6.5		DIM	ST	ST×DIM
Hepatic flows, L/h																	
Plasma	226^b^	224^b^	229^b^	359^a^	14	245^cd^	295^a^	280^ab^	261^abc^	274^abc^	254^bc^	247^bcd^	219^d^	15	< 0.001	0.006	0.58
Blood	312^b^	302^b^	311^b^	501^a^	19	362^b^	419^a^	381^b^	350^b^	363^b^	342^b^	336^bc^	299^c^	20	< 0.001	0.005	0.46
Net hepatic fluxes																	
O_2_, mmol/h	-575^b^	-542^b^	-537^b^	-904^a^	61	− 603	− 695	− 693	− 670	− 681	− 671	− 588	− 516	69	< 0.001	0.32	0.65
CO_2_, mmol/h	490^b^	401^b^	417^b^	764^a^	62	393^b^	572^a^	601^a^	616^a^	589^a^	538^ab^	442^ab^	392^b^	78	< 0.001	0.03	0.22
Insulin, mol/h	−16.3^bc^	−13.7^c^	−23.7^b^	−35.9^a^	3.4	−9.4^cd^	−59.5^a^	− 28.1^b^	−29.4^b^	− 17.9^c^	− 15.9^cd^	− 11.6^cd^	−7.5^d^	3.9	< 0.001	< 0.001	0.003
Urea, mmol/h	40.5^b^	36.9^b^	33.5^b^	75.5^a^	4.2	35.8	37.4	55.5	52.4	54.8	49.5	44.6	42.9	5.4	< 0.001	0.09	0.12
Essential AA, g/d																	
Lys	−3.3	−4.9	− 5.5	− 3.9	0.9	−2.5^c^	−6.3^ab^	−8.0^a^	−6.4^ab^	− 2.1^c^	− 4.4^bc^	− 3.4^bc^	− 2.1^c^	1.3	0.12	< 0.001	0.09
Met	(1.0)	(0.5)	(0.1)	(0.6)	0.6	1.4	(0.0)	(0.3)	(−0.9)	(0.1)	1.2	1.7	(1.0)	0.8	0.68	0.20	0.35
Thr	−1.9	−3.5	−2.9	−4.0	0.9	− 3.1^abc^	−5.6^a^	− 5.0^ab^	− 1.9^c^	− 0.6^c^	− 2.9^abc^	− 3.1^abc^	− 2.5^bc^	1.1	0.52	0.03	0.21
Trp	−2.7^b^	−3.4^b^	− 2.4^b^	−6.7^a^	0.4	− 3.1	− 4.2	− 4.3	− 3.9	− 4.2	− 3.9	− 4.0	−2.5	0.6	< 0.001	0.26	0.76
Ile	(− 1.3)	− 3.7	−2.0	(− 0.6)	0.9	1.0^b^	− 3.2^a^	− 3.5^a^	− 2.7^a^	0.0^b^	−3.3^a^	−1.9^ab^	− 1.5^ab^	1.1	0.05	0.003	0.14
Leu	−2.7	−4.3	−3.0	(−0.7)	1.0	(0.5^d^)	−6.3^ab^	−6.6^a^	−3.3^c^	(−0.6^cd^)	−1.7^abc^	−2.2^abc^	−1.5^bcd^	1.5	0.07	< 0.001	0.006
Val	(1.5)	−2.8	(−0.5)	(1.4)	1.3	2.7	(0.7)	−2.3	(0.1)	−2.5	(−0.8)	2.2	(−0.7)	2.0	0.05	0.24	0.12
His	−5.4^b^	−5.3^b^	−5.1^b^	−9.1^a^	1.2	−6.6	−7.9	− 5.4	−4.5	−8.9	− 5.1	− 4.6	− 6.6	1.6	0.02	0.20	0.35
Phe	−14.9^b^	−15.8^b^	−9.6^c^	−22.5^a^	1.5	−10.0^c^	− 17.9^ab^	− 20.0^a^	−19.6^a^	− 16.7^ab^	−16.5^ab^	− 13.9^bc^	−11.2^c^	1.5	< 0.001	< 0.001	0.25
Non-essential AA, g/d																	
Ala	−49.5^b^	−51.5^b^	−52.5^b^	−86.3^a^	5.8	−52.5^cd^	−67.4^ab^	−76.1^a^	− 66.1^b^	− 59.9^bc^	− 59.0^bc^	−51.3^cd^	−47.4^d^	5.4	< 0.001	< 0.001	< 0.001
Asn	−4.3^b^	−4.9^b^	−3.2^b^	−8.8^a^	0.8	−3.2^d^	−6.0^b^	−8.6^a^	− 6.5^b^	−5.4^bc^	−5.0^bcd^	− 4.1^cd^	−3.6^cd^	0.7	< 0.001	< 0.001	0.02
Asp	−3.4^b^	−3.5^b^	−2.9^b^	−6.6^a^	0.6	− 2.4^b^	−5.7^a^	−6.6^a^	−4.1^b^	− 3.2^b^	− 3.7^b^	−3.6^b^	− 3.6^b^	0.8	< 0.001	0.003	0.22
Cys	−5.6	−7.1	−4.0	− 7.1	1.0	−2.9^c^	−9.9^a^	− 7.8^ab^	− 5.6^bc^	−4.3^bc^	− 6.4^bc^	−5.2^bc^	− 5.3^bc^	1.2	0.05	< 0.001	0.82
Glu	139.4^bc^	130.2^c^	171.8^ab^	177.6^a^	12.8	131.4^b^	152.3^ab^	174.3^a^	162.8^ab^	160.7^ab^	168.6^a^	147.8^b^	140.0^b^	10.9	0.007	0.002	0.10
Gly	−30.5^b^	−33.6^b^	−32.2^b^	−56.9^a^	3.6	−46.3	−35.6	− 37.4	− 33.3	−35.1	−40.6	− 37.7	− 40.3	4.3	< 0.001	0.55	0.002
Pro	−9.2^ab^	−14.7^a^	(−5.1^b^)	−13.6^a^	2.8	−9.6	− 13.2	− 11.4	− 13.4	−7.8	− 12.0	−9.5	−8.3	4.0	0.04	0.84	0.09
Ser	−13.6^a^	−12.9^a^	−5.5^b^	− 14.8^a^	2.0	− 5.9^d^	−16.7^ab^	− 19.1^a^	−14.7^b^	− 10.9^c^	−11.0^c^	−7.5^d^	−7.8^cd^	1.7	0.007	< 0.001	0.001
Tyr	−12.6^b^	−13.3^b^	− 10.3^b^	−21.5^a^	1.3	− 12.3^b^	−16.6^a^	− 18.2^a^	−15.3^ab^	− 13.7^b^	−15.0^ab^	−12.3^b^	− 12.1^b^	1.5	< 0.001	0.01	0.42
Gln	−12.1^b^	−12.1^b^	−32.0^a^	−28.9^a^	4.1	−18.7	−29.3	−17.4	−19.2	− 27.4	− 19.4	− 24.1	−14.8	5.2	< 0.001	0.26	0.98

^a–d^Within a row and within a main effect (DIM, ST), means with different letters differ

^1^Values presented in parenthesis are not significantly different from zero. ^2^*DIM* days in milk, *ST* sampling time

### Hepatic extraction

The hepatic extraction of most AA was rather low (− 4% to 5%), but extraction of His (7%), Phe (10-14%) and most non-essential AA was higher, whereas Glu had a highly negative extraction (indicating release) and was most negative at + 3 DIM. The hepatic extraction of Lys (*P* < 0.001), Ile, Val, Cys, Glu, Ser, and Gln were influenced by DIM (*P* < 0.05; Table 7), while the hepatic extraction of Lys, Ile, Val, Cys, Glu, Gly, and Ser were affected by ST relative to feeding (*P* < 0.05). In addition, an interaction between DIM and ST relative to feeding was observed for Ala (*P* < 0.001), Asn (*P* = 0.004), Gly (*P* = 0.005), and Ser (*P* = 0.02). Hepatic extraction of Lys, Ile and Val were greater at − 3 and + 3 DIM as compared with − 10 and + 17 DIM, it was greater pre partum for Cys and Ser as compared with + 3 and + 17 DIM, and hepatic extraction was greatest at + 3 DIM for Gln as compared with the three other stages.

**Table 7:** Hepatic extraction of amino acids in sows during late gestation and in early and peak lactation^1^

Item	DIM^2^				SEM	ST^2^								SEM	*P*-value		
	−10	− 3	3	17		−0.5	0.5	1.5	2.5	3.5	4.5	5.5	6.5		DIM	ST	ST×DIM
Essential AA, %																	
Lys	2.8^bc^	5.3^a^	4.5^ab^	1.4^c^	0.7	1.2^d^	4.5^ab^	6.1^a^	3.9^abc^	2.0^cd^	4.1^abc^	3.6^abcd^	2.6^bcd^	1.0	< 0.001	0.008	0.09
Met	−4.1	−4.1	(−0.7)	−1.8	2.4	−7.0	−3.1	(− 0.8)	1.8	1.1	−4.0	−6.9	−2.5	3.0	0.64	0.11	0.13
Thr	1.6	2.9	2.7	2.1	0.6	2.1	3.6	3.2	1.8	(0.6)	2.6	2.5	2.5	0.8	0.32	0.09	0.22
Trp	3.5	4.4	3.6	4.4	0.5	3.1	4.3	3.6	3.9	4.6	4.6	4.3	3.5	0.7	0.39	0.42	0.59
Ile	(0.7^bc^)	2.6^a^	2.3^ab^	(0.2^c^)	0.7	−1.1^c^	2.4^a^	2.5^a^	2.0^ab^	(0.0^b^)	2.8^a^	1.6^ab^	1.4^ab^	0.9	0.03	0.006	0.54
Leu	1.0	2.8	2.0	1.3	1.1	−1.6	3.1	3.4	2.4	(0.9)	2.2	2.9	(0.8)	1.5	0.42	0.05	0.55
Val	−0.7^b^	1.4^a^	(0.3^ab^)	(−0.6^b^)	0.6	−2.2^b^	(0.2^a^)	1.2^a^	(0.3^a^)	1.2^a^	(0.8^a^)	(−0.5^ab^)	(0.0^ab^)	0.9	0.03	0.02	0.41
His	7.1	7.2	7.0	6.7	1.2	7.6	7.4	5.6	6.0	8.5	6.4	5.2	9.2	1.7	0.98	0.53	0.79
Phe	12.5	13.9	10.4	12.3	1.2	10.4	12.3	12.9	13.3	12.4	13.5	12.3	11.3	1.3	0.07	0.40	0.37
Non-essential AA, %																	
Ala	16.6	16.3	19.7	19.4	1.8	19.4	17.2	17.8	18.1	16.8	18.1	17.5	19.2	1.8	0.05	0.19	< 0.001
Asn	16.4	20.5	23.4	21.2	1.9	20.4	21.8	23.2	18.8	17.7	19.4	20.2	21.3	2.2	0.12	0.50	0.004
Asp	8.1	8.8	7.3	8.7	1.5	4.8	10.9	10.0	6.9	6.2	9.1	8.5	9.3	1.8	0.86	0.07	0.28
Cys	4.3^a^	5.0^a^	2.4^b^	3.5^ab^	0.6	2.4^c^	5.3^a^	4.4^ab^	3.3^bc^	3.3^bc^	4.2^ab^	3.6^abc^	3.9^abc^	0.7	0.02	0.01	0.91
Glu	−72.2^b^	−71.8^b^	−85.8^a^	−63.8^b^	5.2	−73.7^ab^	−65.8^c^	−73.8^ab^	−72.0^ab^	−71.8^b^	−77.5^ab^	−71.0^b^	−81.7^a^	4.6	0.02	0.005	0.13
Gly	8.9	9.2	9.6	10.3	0.9	10.7^ab^	9.0^bc^	8.9^bc^	8.2^c^	8.2^c^	9.5^bc^	9.8^abc^	11.6^a^	1.1	0.38	0.03	0.005
Pro	3.1	4.2	2.5	2.6	0.7	3.0	3.2	3.6	3.4	1.9	3.6	3.1	3.2	1.0	0.23	0.76	0.16
Ser	13.4^a^	12.6^a^	6.7^b^	9.0^ab^	1.7	6.0^d^	13.1^ab^	14.5^a^	12.3^ab^	9.4^cd^	10.7^bc^	8.2^cd^	9.1^cd^	1.5	0.02	< 0.001	0.02
Tyr	10.0	10.8	10.3	10.1	0.9	9.4	10.9	11.0	9.9	9.5	10.7	9.5	11.5	1.1	0.85	0.46	0.49
Gln	4.6^b^	5.9^b^	10.9^a^	6.3^b^	1.6	7.8	8.1	5.3	5.9	8.0	6.2	8.4	5.7	1.8	0.03	0.56	0.61

^a–d^Within a row and within a main effect (DIM, ST), means with different letters differ

^1^Values presented in parenthesis are not significantly different from zero. ^2^*DIM* days in milk, *ST* sampling time

### Heat production

Heat production in PDV and liver and total sow HE as well as the ratio of PDV to total HE were significantly affected by DIM (*P* < 0.001, Table 8). Heat production in PDV and liver at peak lactation were higher than that at other stages (*P* < 0.05). Total HE in lactation was higher than that in late gestation (*P* < 0.05). The RQ of PDV and liver did not differ across DIM (*P* > 0.05).

**Table 8:** Heat production and respiratory quotient of portal drained viscera and liver in sows during late gestation and in early and peak lactation

Item	DIM^1^				SEM	*P*-value
	−10	−3	3	17		
HE^1^, MJ/d						
PDV^1^	6.20^b^	6.02^b^	5.58^b^	9.77^a^	0.25	< 0.001
Liver	7.21^b^	6.66^b^	6.54^b^	11.91^a^	0.83	< 0.001
Total HE	31.55^b^	31.75^b^	39.77^a^	41.52^a^	0.80	< 0.001
PDV, %	18.10^b^	17.54^b^	10.63^c^	29.13^a^	2.29	< 0.001
Liver, %	16.82	22.50	16.24	32.98	5.04	0.104
RQ^1^						
PDV	1.16	1.20	1.21	1.29	0.04	0.30
Liver	0.83	0.75	0.71	0.75	0.05	0.45

^a–c^Within a row and within a main effect (DIM), means with different letters differ

^1^*DIM* days in milk; *HE* heat production; *PDV* portal drained viscera; *RQ* respiratory quotient

### Correlation between insulin and amino acids

Correlation analysis was performed to evaluate the potential link between insulin and amino acids. The net portal flux of insulin was positively correlated with all net portal fluxes of AA (*P* < 0.05; data not shown), except for Cys and Glu. The net hepatic flux of insulin was positively correlated with most net hepatic fluxes of AA (*P* < 0.05), and it was negatively correlated with the net hepatic flux of Glu (*P* < 0.001).

### Essential amino acid efficiencies

**Fig. 4:**
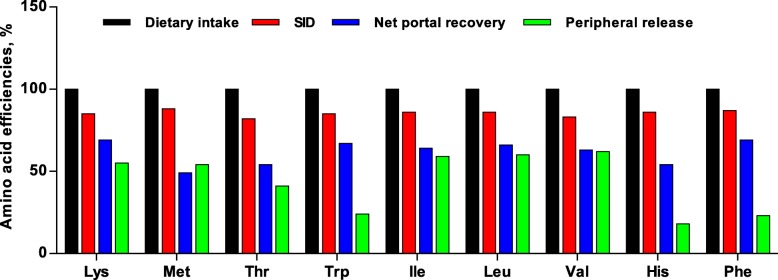
The average efficiencies of essential amino acid in sows during late gestation and lactation: standardized ileal digestibility (SID), net portal recovery, and peripheral release (net splanchnic recovery) of essential amino acid refer to dietary intake

The standardized ileal digestibility (SID) of essential AA were within 82-88% (Fig. 4). Net portal recovery of most essential AA were within 63-69%, while Met, Thr, and His were clearly lower (49-54%). Net splanchnic recovery of most essential AA were within 41-62%, while Trp, His, and Phe were clearly lower (18-24%).

## Discussion

It has been demonstrated that the degree of dietary AA being utilized in first pass by the intestine and liver will determine the availability for the support of peripheral tissue metabolism [24]. Physiological processes rapidly change during the transition and lactation period, thus a dynamic blood flow study is needed to reveal the efficiencies of AA utilization across the PDV and liver in sows.

### Limiting AA in the diet

The dietary lysine concentration in the present study was 7.87 g SID Lys/kg DM equivalent to 7.18 g SID Lys/kg feed. In a new Danish study it was recently demonstrated that the Lys required to maximize average daily litter gain recently was reported to be 8.11 g SID Lys/kg [4], indicating that the sows in the present study most likely was undersupplied with lysine, although it should be noted that the sows in the present study suckled 12 piglets whereas sows in the study by Hojgaard and co-workers suckled 14 piglets [4]. In support of this, the postprandial change in arterial Lys concentration and the low hepatic extraction of Lys at peak lactation compared with early lactation, suggest that dietary supply of Lys was likely the limiting factor for milk production at peak lactation. Compared with the new Danish recommendations [25], the sows in the present study were supplied excess dietary essential SID AA relative to their requirement when expressed relative to lysine (current recommendation given in parenthesis): Met 32% (31%), Thr 72% (65%), Trp 25% (20%), Ile 93% (56%), Leu 157% (108%), Val 105% (69%), His 53% (36%), and Phe 111% (55%). Interestingly, the hepatic extraction rate decreased from 4.5% in early lactation to 1.4% at peak lactation, which suggest that AA other than Lys or maybe energy was limiting sow performance in early lactation. In support of this, Huber et al. observed a substantial decrease in pre-prandial serum concentration of Lys from early to peak lactation [26], indicating a much greater drainage of lysine from the plasma pool to the mammary glands for milk production at peak lactation as compared with early lactation [7, 27].

### Feed intake, digestibility and net portal fluxes

The ATTD of DM, GE, nitrogen, fat and crude fiber decreased in the present study for sows fed a wheat-barley-soybean meal-based diet as feed intake level increased and was mainly explaining changes in net portal fluxes, whereas no evidence of changes in net portal recoveries across DIM was found. The result is in agreement with the observations reported that the ATTD of DM, GE, organic matter, and fat diminished with increasing feeding levels for pigs fed a cornstarch-SBM-based diet [28]. One possible reason is that the enhanced feed intake led to a faster passage rate in the gastrointestinal tract, allowing less time for fermentation in the hindgut, thus reducing the digestibility of all nutrients and energy [29]. Another possible explanation could be that the influence of endogenous excretions may be changed according to feeding levels [30]. However, other researchers observed no differences of ATTD of energy and nutrients in response to feeding levels [31, 32], while Dourmad and co-workers even found that ATTD of DM, organic matter, and GE increased linearly with the increase of energy intake [33]. The inconsistent findings concerning the effect of feeding levels on digestibility of energy and nutrients could most likely be attributed to differences in dietary composition and age of pigs.

The NPF of nutrients were considerably influenced by DIM in the current study. These changes were essentially reflected by differences in feed intake and levels of digested nutrients. All NPF of AA were positive and followed the same pattern as changes in feed intake, except for Glu. Compared to pre partum levels, net portal uptake of AA at + 3 DIM were reduced (3-63% lower) and almost doubled (40-100% higher) at + 17 DIM. It was clearly showing that an increase in feed intake increased net portal uptake of AA, associating with increased milk production in peak lactation [34]. The negative net portal flux of Glu indicated oxidation or metabolism of Glu by the intestinal mucosa. Similarly, a previous study showed that almost all Glu and Asp in the diet are catabolized by the small intestinal mucosa in the first pass [35]. Although Glu and Asp were the most abundant amino acids in the diets (Table 1), very small amounts appeared in the portal blood due to the extensive oxidation by the small-intestinal epithelial cells [36, 37]. Among the non-essential AA, a very low net portal recovery of Asp and Glu was observed as well as a high net portal recovery of Ala and Gly which both exceeded 100%. These high portal recoveries are only possible if other AA are transaminated in the intestinal mucosa during the absorptive process [38, 39].

Accurate AA efficiency values are key to designing nutritional models for prediction of AA requirement and may also help to improve feed formulation during the optimization of these [40]. In this study, the net portal recovery of most essential AA were within 63-69%, while Met, Thr, and His were clearly lower (49-54%), and none of these differed from late gestation to peak lactation. This is in agreement with previous studies, which reported that 30-50% of essential AA in the diet of growing pigs may be undigested or catabolized by the small intestine in first-pass metabolism [24, 41]. These results indicate that intestinal metabolism of dietary AA alters both the amount and pattern of AA absorbed into the portal circulation. The net portal recovery of essential AA was constant across these physiological stages and hence not related to changes in feed intake or physiological processes like fetal and mammary growth (in late gestation) or milk production after parturition. The net portal recoveries of Lys and Val in peak lactation is comparable with the maximum biological efficiency value of them estimated by Zhang et al. [5]. However, the net portal recoveries of the remaining essential AA were substantially lower in this study than the MBEV reported by Zhang and co-workers. This discrepancy most likely arise from the differences in the dietary formulation, as the feed in the present study was formulated without crystalline AA, whereas the study by Zhang et al. [5] extensively used crystalline AA to obtain a near ideal AA profile. Sulfur containing AA (Met+Cys) and His had lower net portal recovery than the remaining essential AA. Sulphur containing AA (Met+Cys) and aromatic AA (Phe, Tyr, Trp and His) play important roles in intestinal mucosal growth associated with a regulated redox status, intestinal epithelial cell function [42] and in acute phase responses [43], and deserve to be studied further around parturition where the sow secrete substantial amounts of immunoglobulins via colostrum.

### Liver metabolism of amino acids

The liver plays an essential role in intermediary metabolism of sows during transition and lactation, and the metabolic burden of the liver increased considerably from late gestation to peak lactation. Amino acid metabolism in liver is a function of AA requirements of peripheral tissues and net portal supply of AA. In the current study, the net hepatic fluxes of Met and Glu were positive, indicating liver release. It is striking that Glu is highly abundant both in feed and in milk [44], but Glu is not at all net absorbed from the GI-tract to the portal blood, and consequently the liver must synthesize substantial amounts of this particular AA. Net hepatic fluxes of Ala and Gly were always negative (indicating hepatic uptake), but they were similar pre partum and at + 3 DIM, whereas they were substantially increased (71% and 78%, respectively) at + 17 DIM. Glycine and Ala are the two most important AA for recirculating nitrogen and C-2 (Gly) or C-3 (Ala) carbon skeletons back to the liver from peripheral tissues [45]. Glutamate (C-5 molecule) is released from the liver as a result of its endogenous synthesis, therefore, there was a significant transfer of this AA from the liver to other organs, in particular the mammary gland [27]. Moreover, the present study showed a low hepatic extraction of BCAA being approximately 0 to 2.8%. It indicated that the capacity of BCAA metabolism in the liver was limited and at peak lactation the net hepatic uptake did not differ from zero for any of the three BCAA. This is consistent with previous study, which reported that pigs had a low activity of hepatic BCAA transaminase [46]. In addition, the net splanchnic recovery of most essential AA were within 41-62% (Fig. 4), but Trp, His, and Phe were clearly lower (18-24%). The low net splanchnic recoveries of these AA indicate a high hepatic oxidation. Hepatic uptake of AA peaked around 1.5 h after feeding (range 0.5-2.5 h) and concomitantly the hepatic release of urea tended to peak, indicating that except for Met, Val, and Glu, a substantial loss of both essential and non-essential AA occur during the first few hours after feeding. These findings suggest that the liver is able to judge whether excessive amounts of AA are present in the circulating blood, possibly by evaluating critical concentrations of each specific AA in portal blood and based on that decide whether the AA should be oxidized. If that working hypothesis is true, our data suggest that more frequent meals of smaller portion size would be favorable to minimize the postprandial increases in portal blood and thereby increase the efficiency of utilizing dietary AA for milk production.

Urea is synthesized in the urea cycle by the liver. In line with that, the net hepatic flux of urea was substantially positive, indicating urea release from the liver amounting to 36.9-40.5 mmol/h pre partum, and increased two fold to 75.5 mmol/h at 17 DIM. To cover the increased demand for AA for synthesis of milk protein, greater amounts of AA were net absorbed from PDV (due to increased feed intake), but more AA were concomitantly being oxidized by the liver [47]. This results in a concomitant increase in urea synthesis to remove the extra AA nitrogen by liver, as illustrated by increased arterial urea concentration in peak lactation. However, a negative net portal flux of urea was observed at all times (− 2.6 to − 21.1 mmol/h), showing that part of the urea was recycled to the gastrointestinal tract where it may become incorporated into microbial biomass [48]. Without doubt, the hepatic oxidation of AA could be minimized by including crystalline AA to avoid great excess of certain dietary AA, as has recently been demonstrated by Zhang et al. [5].

### Heat production in PDV and liver

The portal blood flow could reflect a circulatory response to gastrointestinal function related to the process of digestion and absorption [49]. Indeed, a positive relationship between feed intake and blood flow has been reported previously [50, 51]. Moreover, the oxygen consumption clearly increased by the PDV and liver at peak lactation. The increase in oxygen consumption by the PDV was proposed to result partially from an increase in net nutrient absorption across the PDV, whereas the increase in oxygen consumption by the liver most likely reflects an increased metabolic activity, especially at peak lactation. In support of this, Freetly and Ferrell reported that oxygen consumption by the PDV and milk production were positively related to digestible energy intake in lactating ewes [52]. Consistent with these observations, the heat production by PDV and liver at peak lactation were higher than that at other stages. Moreover, the heat production by PDV relative to total HE markedly changed especially from early (11%) to peak lactation (29%), whereas the heat production by liver relative to total HE was doubled from 16% to 33% from early to peak lactation (although it should be noted that the hepatic values only tended to differ (*P* = 0.10). Similarly, Stoll and Burrin reported that the PDV represented 4% to 6% of body weight in pigs, but accounts for 20% to 35% of the O_2_ consumption and total heat production [53]. Taken together, the heat production by PDV and liver are important for the total energy requirement of the animals, while the proportion of PDV and liver heat production relative to the total sow heat production is variable because of differences in feed intake, physical activity, and physiological stage.

### Insulin secretion

It is well known that insulin is the central hormone regulating glucose homeostasis, mainly by regulating plasma glucose concentration in the portal blood. However, amino acids also play an important role in regulating insulin secretion [54]. In this study, arterial insulin concentrations markedly increased from − 3 DIM to + 3 DIM, even though feed intake and hence net portal uptake of glucose (data not shown) and AA decreased, and the higher level of arterial insulin was maintained until peak lactation. The increased proportional secretion of insulin at + 3 DIM could be caused by the catabolic state of the sow. Similarly, Theil et al. [55] found that the apparent insulin secretion was much larger and did not correlate to net glucose absorption in growing pigs after a 14 h overnight fast as compared with pigs fed every 5 h. Pere and Etienne reported that the development of insulin resistance appeared at the end of gestation, and further accentuated during lactation accompanying with higher energy intake [56]. These results may suggest that sows from late gestation to early lactation develop insulin resistance, which favors the nutrient uptake to the mammary gland as milk yield increase [57], although this study was not designed for detecting insulin resistence. Postprandially, most net portal and net hepatic fluxes of AA significantly fluctuated accompanying with the similar change of insulin concentration. In fact, a positive Pearson correlation coefficient was found between most net portal or hepatic fluxes of AA and net insulin fluxes, respectively (data not shown). In line with this, van Loon et al. reported that insulin responses were positively correlated with plasma AA concentrations [58].

## Conclusions

Our study revealed that the net portal recoveries differed for the essential AA but were constant across physiological stage (from − 10 to + 17 d relative to parturition) in spite of great changes in feed intake, nutrient digestibility, and sow physiology. Net portal uptake of AA peaked at 2.5 h after feeding and coincided with the greatest hepatic oxidation of AA. The study suggests that more frequent meals with smaller portion size may be an efficient way to minimize AA oxidation and to increase utilization of dietary AA in the future. Also, the study indicate that it is beneficial to add crystalline AA to lactation diets for sows to minimize the excess of dietary AA. Moreover, the study indicated that sulphur containing AA and histidine act differently than other essential AA and deserve to be further studied. Finally, the study indicated that dietary lysine most likely was the limiting AA at peak lactation.

## Data Availability

All data generated or analyzed during this study are included in this published article.

## Abbreviations

AA: Amino acids
ATTD: Apparent total tract digestibility
DIM: Day in milk
DM: Dry matter
GE: Gross energy
HE: Heat production
*p*AH: *Para*-amino hippuric acid
PDV: Portal-drained viscera
RQ: Respiratory quotient
